# Muito além de uma história das leishmanioses no Novo Mundo

**DOI:** 10.1590/S0104-59702023000100056

**Published:** 2023-10-23

**Authors:** Denis Guedes Jogas

**Affiliations:** i Programa de Pós-graduação em História das Ciências e da Saúde Casa de Oswaldo Cruz Fiocruz Rio de Janeiro RJ Brasil denis.jogas@hotmail.com Programa de Pós-graduação em História das Ciências e da Saúde/Casa de Oswaldo Cruz/Fiocruz.Rio de Janeiro – RJ – Brasil denis.jogas@hotmail.com

Em dezembro de 2022 foi lançado o segundo volume da trilogia *Uma história das leishmanioses no Novo Mundo*, fruto de parceria entre as editoras Garamond e Fiocruz. Com narrativa densa e envolvente, o pesquisador Jaime Larry Benchimol, titular da Casa de Oswaldo Cruz/Fiocruz, e Cláudio de Oliveira Peixoto, analista em gestão de saúde do Instituto Leônidas e Maria Deane/Fiocruz-AM e doutorando do Programa de Pós-graduação em Saúde Global e Sustentabilidade da Universidade de São Paulo, transportam-nos para a Amazônia, revelando as fascinantes e específicas maneiras pelas quais as pesquisas realizadas na região contribuíram para a construção de conhecimentos sobre as leishmanioses em escala global entre 1960 e os primeiros anos do século XXI.

Com prefácio assinado pelo historiador das ciências Nelson Sanjad e oito capítulos, os autores não se limitaram à análise do objeto central da obra, isto é, a história das leishmanioses, mas também lançaram luz sobre pesquisas desenvolvidas acerca de várias enfermidades como malária, filariose, tripanossomíases, arboviroses, entre outras. Além disso, as trajetórias profissionais dos cientistas e de suas respectivas instituições são pontos fortes do trabalho. Os personagens abordados no livro têm um papel importante nessa história, e a obra apresenta uma visão ampla e dinâmica da pesquisa científica na área de doenças tropicais.

No primeiro volume, Benchimol e Jogas Junior (2020) demonstraram que, na primeira metade do século XX, a rede global de pesquisas sobre as leishmanioses foi orquestrada pela Société de Pathologie Exotique e por seu fundador e primeiro presidente, Alphonse Laveran, sendo os médicos sul-americanos personagens essenciais na caracterização das leishmanioses do Novo Mundo como doença humana singular e própria dessa região. No novo volume, é dada atenção especial ao papel desempenhado pela London School of Hygiene and Tropical Medicine e pelo parasitologista britânico Percy Cyril Claude Garnham na construção de uma nova rede centrada em nova abordagem, menos antropocêntrica e com maior interesse pelas zoonoses. Essa rede tornava-se cada vez mais densa e policêntrica (no Brasil, inclusive) à medida que grandes projetos de infraestrutura adentravam territórios inabitados e/ou regiões interioranas, alterando drasticamente o ambiente e criando, assim, condições propícias à circulação de patógenos – antes limitados aos animais vetores silvestres – nos ambientes modificados pelo homem, um novo hospedeiro vertebrado.

O início da narrativa se estrutura em torno da criação da Wellcome Parasitology Unit no âmbito do Instituto Evandro Chagas, em Belém do Pará, e da chegada de Ralph Lainson e Jeffrey Shaw, ambos discípulos de Garnham, para comandá-la. Essa unidade de pesquisa, financiada pelo Wellcome Trust, tinha como objetivo primário o estudo de possíveis hospedeiros silvestres dos parasitos responsáveis pelas múltiplas formas clínicas da leishmaniose tegumentar americana em animais existentes na vasta fauna das florestas tropicais; em especial, na amazônica. Merece menção a envolvente digressão histórica feita por Benchimol sobre a história da fusão da Wellcome Foundation e do Wellcome Trust, a fim de dar aos leitores uma ideia sobre o papel dessa organização no mercado global de fármacos e no financiamento das ciências da vida e da saúde em várias partes do mundo, inclusive no Brasil.

Como é demonstrado por Benchimol e Peixoto, apesar de algumas desavenças no cotidiano do Instituto Evandro Chagas, as profícuas prospecções e pesquisas lá desenvolvidas produziram significativas mudanças na maneira pela qual eram compreendidas as leishmanioses e outras doenças parasitárias na região amazônica, sobretudo a partir da década de 1970, quando os parasitologistas britânicos, em colaboração com pesquisadores da região, como o parasitologista Orlando Rodrigues da Costa e os entomologistas Reynaldo Goubert Damasceno e Habib Fraiha Neto, demonstraram que eram muito mais diversificadas do que se pensava as populações de parasitos, vetores e hospedeiros das leishmanioses, um complexo de doenças diferentes clínica e epidemiologicamente. Assim, desbancaram de vez a já abalada soberania da *Leishmania braziliensis* como único agente etiológico das diferentes formas da leishmaniose tegumentar americana. Um conceito sedimentado na década de 1930, como mostram Benchimol e Jogas Junior (2020).

Benchimol e Peixoto (2022) também trazem à tona as complexas interações entre o recrudescimento de doenças como as leishmanioses e a malária e os grandes projetos desenvolvimentistas implementados pelo governo federal na região, durante o regime militar. A obra examina em detalhes a relação entre urbanização, surtos e expansão geográfica das leishmanioses tegumentares e a criação da Zona Franca de Manaus, a exploração de minério em Carajás e Pitinga, o projeto de produção de celulose em Jari e a construção das rodovias Transamazônica e Belém-Brasília, da hidrelétrica de Balbina e do gasoduto Coari-Manaus. Os autores revelam a consolidação concomitante de uma *expertise* local por pesquisadores ligados à tradição parasitológica brasileira como os paraenses Heitor Vieira Dourado e Carlos Borborema, fundadores da Clínica de Moléstias Tropicais, embrião da atual Fundação de Medicina Tropical Doutor Heitor Vieira Dourado, e estrangeiros associados a instituições amazonenses, como Jorge Ramon Arias e Toby Barret no Instituto Nacional de Pesquisas da Amazônia. Esses e outros cientistas desempenharam papéis relevantes não apenas no enfrentamento dos surtos epidêmicos ensejados pelos projetos de desenvolvimento, que deveriam trazer modernização à região, como também na descrição de novas espécies de parasitos e vetores das *Leishmania* e de outros parasitos. Produziram ou propuseram esses pesquisadores inovações no tocante a prevenção, tratamento e controle por meio da aplicação de novíssimas técnicas biomoleculares que foram essenciais para o conhecimento da epidemiologia, da ecologia e dos fatores de risco das leishmanioses na Amazônia.

O livro, portanto, apresenta uma perspectiva dinâmica sobre a pesquisa científica na região amazônica e as complexas interações entre saúde pública, desenvolvimento econômico e transformações socioambientais. Configura-se como excelente oportunidade não apenas para aqueles que querem conhecer melhor a história das leishmanioses na região Norte do país, mas também para quem deseja compreender o cotidiano e as dinâmicas de pesquisas nas instituições amazônicas, assim como as redes e os contatos por elas estabelecidos com centros de pesquisas congêneres em outras regiões do Brasil e do exterior. Afinal, como Jaime Benchimol sempre gostou de frisar, a história de uma doença nunca é apenas a história só de uma doença.
